# Twelve-Month Outcomes of iStent Infinite in Severe Open-Angle Glaucoma Compared with iStent Inject W in Mild-to-Moderate Disease

**DOI:** 10.3390/jcm15166396

**Published:** 2026-08-19

**Authors:** Sangwon Han, Sun Woong Kim

**Affiliations:** Department of Ophthalmology, Yonsei University Wonju College of Medicine, 20 Ilsan-ro, Wonju 26426, Gangwon-do, Republic of Korea; sw7596@naver.com

**Keywords:** open-angle glaucoma, minimally invasive glaucoma surgery, iStent inject W, iStent infinite, intraocular pressure, disease severity

## Abstract

**Background/Objectives**: The efficacy of two-stent implantation with iStent inject W is well established for mild-to-moderate open-angle glaucoma (OAG), whereas evidence for three-stent implantation with iStent infinite in severe OAG remains limited. We compared 12-month outcomes of a severity-based surgical strategy, using two stents in mild-to-moderate OAG and three stents in severe OAG. **Methods**: This retrospective study included 100 eyes with OAG that were followed for at least 12 months after trabecular micro-bypass stent implantation. Eyes with mild-to-moderate disease, defined as visual field mean deviation (MD) of −12 dB or better, received two stents (iStent inject W), whereas eyes with severe disease, defined as MD worse than −12 dB, received three stents (iStent infinite). Outcomes included intraocular pressure (IOP), glaucoma medication burden, visual field parameters, corneal endothelial cell density (ECD), surgical success, and additional glaucoma surgery. **Results**: Baseline IOP and the number of glaucoma medications were higher in the three-stent group. At 12 months, mean IOP was similar between groups. Although the three-stent group showed a greater unadjusted reduction in IOP, this difference was not significant after adjustment for baseline IOP using analysis of covariance. Linear mixed-model analysis also showed no significant group effect or time-by-group interaction. Glaucoma medications decreased significantly in both groups, with a similar mean reduction, although the three-stent group had a greater medication burden at 12 months. Changes in visual field parameters and corneal ECD did not differ significantly between groups. Surgical success and the need for additional glaucoma surgery were also not significantly different between groups. **Conclusions**: In this retrospective cohort, three-stent implantation in severe OAG was associated with 12-month IOP outcomes similar to those observed in the two-stent group with mild-to-moderate OAG, although this was achieved with a greater residual medication burden. This approach appears feasible for patients with severe OAG.

## 1. Introduction

Glaucoma is a progressive optic neuropathy that causes irreversible visual field loss, and lowering intraocular pressure (IOP) is the only proven means of slowing disease progression [1,2]. Traditional filtration surgery achieves a substantial reduction in IOP but is limited by a high risk of postoperative complications and an unpredictable wound healing response [3]. These drawbacks prompted the development of minimally invasive glaucoma surgery, which directly bypasses the trabecular meshwork, the primary site of aqueous outflow resistance. Compared with filtration surgery, this approach offers a more favorable safety profile and more predictable IOP reduction while preserving conjunctival and scleral tissue [4,5].

Among the devices used in these procedures, the iStent inject W, which delivers two titanium stents into Schlemm’s canal, has well-established clinical efficacy and safety in patients with mild-to-moderate open-angle glaucoma (OAG) [6,7,8]. The iStent infinite, a newer device that delivers three stents, was introduced for patients with more advanced disease or with prior treatment failure [9,10].

The physiological rationale for using three stents in patients with severe OAG is related to the anatomical variability of the distal outflow system [5]. As the disease progresses, outflow resistance within the trabecular meshwork and beyond Schlemm’s canal increases in a more complex manner [11]. Therefore, implanting three stents at more widely distributed locations increases the probability of accessing patent collector channels and the episcleral venous network [12,13].

While the anatomical and physiological rationale is well-established, few clinical studies have evaluated the severity-stratified use of iStent inject W and iStent infinite in distinct patient cohorts [9,14,15]. Rather, the existing literature has primarily focused on the isolated effects of each device, or analyzed them using heterogeneous patient groups without clear baseline severity criteria [16,17,18].

Therefore, this study evaluated postoperative outcomes in patients with mild-to-moderate OAG who received two stents (iStent inject W), and in patients with severe OAG who received three stents (iStent infinite) [19]. We further analyzed changes in IOP, topical glaucoma medication use, visual field parameters, and endothelial cell density (ECD) over a minimum follow-up period of 12 months. Through this, we sought to determine whether three-stent implantation in severe OAG could provide effective postoperative IOP control and acceptable short-term safety while monitoring early postoperative visual field changes.

## 2. Materials and Methods

### 2.1. Study Design and Patients

This study was a single-center, retrospective medical record review of 100 eyes that underwent trabecular micro-bypass stent implantation by a single experienced surgeon (S.W.K.) at the Department of Ophthalmology, Yonsei University Wonju Severance Christian Hospital and were followed for at least 12 months. This study adhered to the principles of the Declaration of Helsinki and was approved by the Institutional Review Board of Yonsei University Wonju Severance Christian Hospital (approval number: CR325099). The requirement for informed consent was waived by the board owing to the retrospective nature of the study.

Patients were stratified into two groups by preoperative visual field mean deviation (MD) according to the Hodapp–Parrish–Anderson classification. Sixty-five eyes of patients with mild-to-moderate OAG (MD ≥ −12 dB) received two stents (iStent inject W; Glaukos Corp., San Clemente, CA, USA), and 35 eyes of patients with severe OAG (MD < −12 dB) received three stents (iStent infinite; Glaukos Corp.) [20].

The surgeries were performed either as stand-alone procedures or combined with phacoemulsification. Patients with corneal abnormalities, other types of glaucoma (e.g., neovascular glaucoma, uveitic glaucoma), or a history of retinal surgery or other glaucoma surgeries were excluded from the study. We retrospectively screened consecutive patients who underwent trabecular micro-bypass stent implantation between January 2022 and June 2025. Fifty-eight patients were excluded: 35 had insufficient follow-up, 12 had other types of glaucoma, and 11 had undergone prior glaucoma or retinal surgery. The remaining 100 eyes of 100 patients were included in the final analysis.

### 2.2. Surgical Procedure

All surgeries were performed by a glaucoma surgeon with 5 years of experience performing iStent implantation. In cases combined with cataract surgery (80.0% in the two-stent group and 74.3% in the three-stent group), standard phacoemulsification and intraocular lens implantation through a clear corneal incision were performed first. Subsequently, an ophthalmic viscoelastic device (OVD; Kukje Hyaluronic Acid Eye, Kukje Pharma, Seongnam, South Korea) was injected into the anterior chamber to maintain the angle. The nasal iridocorneal angle was visualized using a gonioprism (Swan–Jacob gonioprism; Ocular Instruments, Bellevue, WA, USA) after the patient’s head and surgical microscope were tilted appropriately (OPMI LUMERA 700; Carl Zeiss Meditec AG, Jena, Germany). Under direct gonioscopic visualization, the injector was advanced through a temporal clear-corneal incision across the anterior chamber toward the nasal angle. The stents were implanted ab interno through the nasal trabecular meshwork into Schlemm’s canal. In the two-stent group, the stents were intended to be implanted in the nasal quadrant, generally at approximately the 2- and 5-o’clock positions in right eyes and the 7- and 10-o’clock positions in left eyes. In the three-stent group, the intended positions were approximately the 1-, 3-, and 5-o’clock positions in right eyes and the 7-, 9-, and 11-o’clock positions in left eyes. Given the retrospective design, the exact positions of individual stents were not routinely recorded; thus, the positions described above represent the intended surgical plan and may have varied slightly among cases. After the stents were positioned, the OVD was removed, and the corneal incisions were sealed by stromal hydration, thus concluding the procedure without sutures. Correct stent positioning was confirmed intraoperatively by gonioscopy in all included eyes. At the 3-month postoperative visit, gonioscopy was performed in all eyes to assess stent position.

### 2.3. Clinical Outcome Measures

The primary efficacy outcome was the change in IOP from baseline to 12 months postoperatively, measured using Goldmann applanation tonometry (Haag-Streit, Köniz, Switzerland) [21]. Postoperative IOP at 1, 3, 6, and 12 months was assessed longitudinally as a supportive analysis. Secondary and exploratory outcomes included changes from baseline to 12 months in the number of topical glaucoma medications, corneal ECD, and visual field parameters obtained with the Humphrey Field Analyzer (Carl Zeiss Meditec, Dublin, CA, USA), including MD, pattern standard deviation (PSD), and visual field index (VFI) [22]. Furthermore, the distribution of IOP at 12 months across predefined categories (<15 mmHg, 15–18 mmHg, and >18 mmHg), the distribution of the number of medications used (0, 1, 2, or 3), surgical success, and the incidence of additional glaucoma surgery were assessed. Safety assessments included transient hyphema, hemorrhage requiring intervention, and early postoperative IOP spikes, defined as an increase of ≥10 mmHg from the preoperative baseline IOP at the 1-week postoperative visit [23]. Surgical success was defined using a composite criterion adapted from the American Academy of Ophthalmology recommendations for reporting clinical endpoints in minimally invasive glaucoma surgery: IOP ≤21 mmHg with ≥20% reduction from baseline without an increase in medications, or a reduction of ≥1 medication from baseline without an increase in IOP [24]. These 12-month IOP outcomes were evaluated under the real-world medication regimen of each patient at the time of measurement, without medication washout, reflecting routine clinical practice.

### 2.4. Statistical Analysis

Statistical analyses were performed using SPSS version 28.0 (IBM Corp., Armonk, NY, USA). Baseline characteristics were compared between the two groups using independent *t*-tests for continuous variables and chi-squared tests or Fisher’s exact tests for categorical variables, as appropriate. Within-group changes in the number of glaucoma medications from baseline to 12 months were assessed with paired *t*-tests.

The between-group comparison of the 12-month change in IOP was considered the primary IOP analysis and was performed using analysis of covariance (ANCOVA) with adjustment for baseline IOP. Longitudinal postoperative IOP measurements at 1, 3, 6, and 12 months were additionally evaluated using a linear mixed model (LMM). The LMM included treatment group, time, group-by-time interaction, baseline IOP, concurrent cataract surgery, and baseline glaucoma medication burden as fixed effects. Full parameter estimates for the ANCOVA and LMM are provided in Supplementary Tables S1 and S2, respectively.

To account for multiple between-group comparisons among the secondary and exploratory outcomes, Holm–Bonferroni adjustment was applied, with unadjusted *p*-values presented in the main tables and Holm-adjusted *p*-values in Supplementary Table S3. As an exploratory sensitivity analysis, the IOP and medication outcomes were also compared separately within the stand-alone and combined cataract surgery subgroups using an independent t-test or Mann–Whitney U test, as appropriate (Supplementary Table S4).

Complete outcome data were available for all included eyes at the evaluated study visits; therefore, no imputation for missing data was required. Residual diagnostics for the ANCOVA and LMM did not indicate serious violations of normality or homoscedasticity assumptions. All statistical tests were two-sided, and *p* < 0.05 was considered statistically significant.

## 3. Results

### 3.1. Baseline Clinical Characteristics

Table 1 details the baseline clinical characteristics of the study patients. All 100 eyes were followed for 12 months. No statistically significant differences were observed between the two-stent and three-stent groups in sex distribution (*p* = 0.719), age (69.96 ± 9.17 years vs. 69.66 ± 11.26 years, *p* = 0.889), or the proportion of concurrent cataract surgery (80.0% vs. 74.3%, *p* = 0.511). Additionally, baseline corneal ECD was comparable between the groups (2403.57 ± 448.76 cells/mm^2^ vs. 2379.95 ± 412.72 cells/mm^2^, *p* = 0.800).

As expected from the study design, MD (−5.83 ± 3.24 dB vs. −18.97 ± 5.61 dB, *p* < 0.001), PSD (4.91 ± 2.86 dB vs. 9.67 ± 2.37 dB, *p* < 0.001), and VFI (89.43 ± 8.83% vs. 47.17 ± 20.08%, *p* < 0.001) differed significantly between the groups, reflecting more advanced disease in the three-stent group. In addition, baseline IOP (15.22 ± 3.82 mmHg vs. 18.03 ± 7.82 mmHg, *p* = 0.046), the number of glaucoma medications (2.02 ± 1.05 vs. 2.83 ± 0.87, *p* < 0.001), and BCVA (logMAR, 0.43 ± 0.33 vs. 0.61 ± 0.43, *p* = 0.023) were significantly worse in the three-stent group.

### 3.2. Changes in Intraocular Pressure and Glaucoma Medications

**Figure 1:**
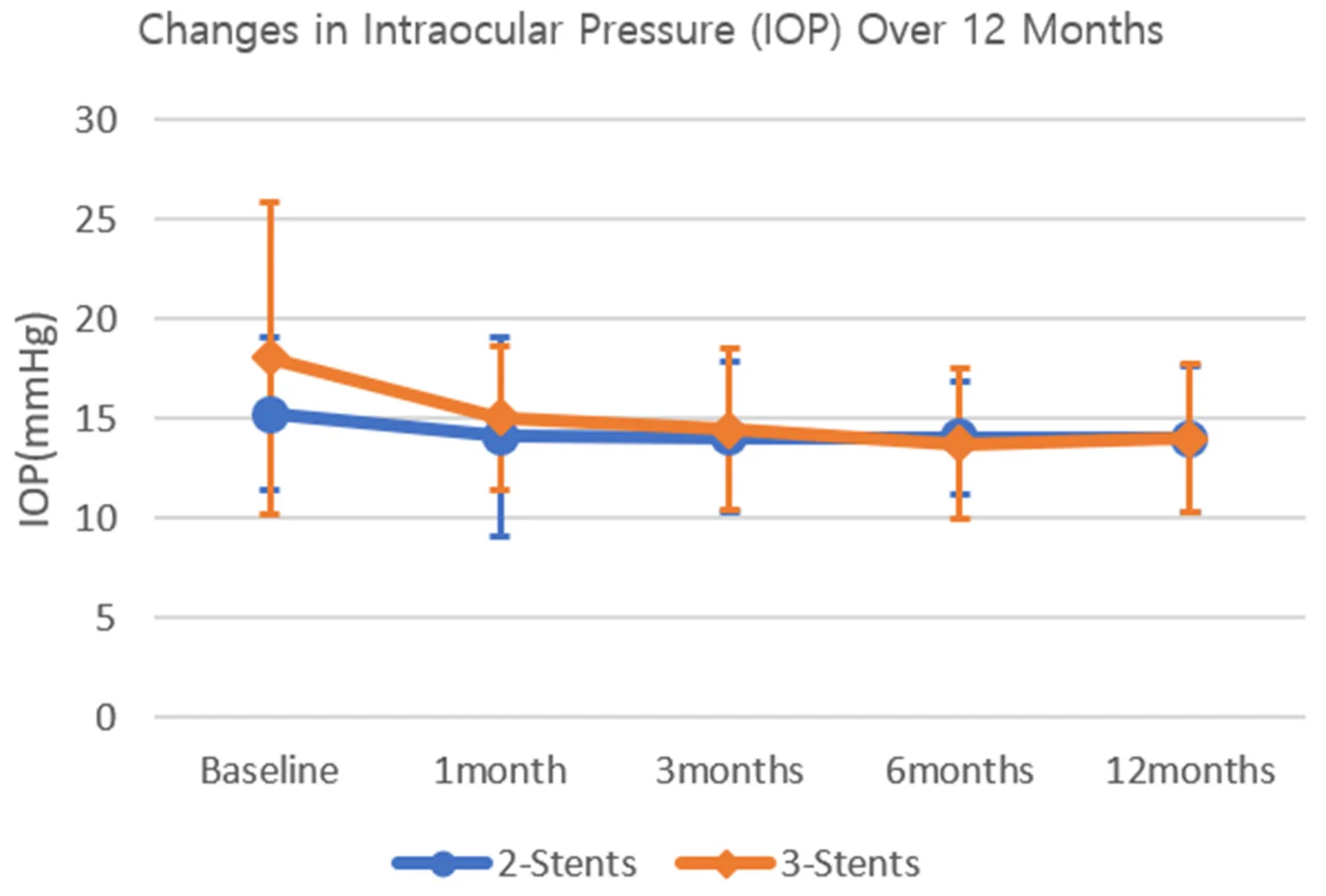
Changes in IOP over the 12-month postoperative period. The line graph shows unadjusted observed mean IOP at baseline and at 1, 3, 6, and 12 months postoperatively for the two-stent group (mild-to-moderate OAG) and the three-stent group (severe OAG); error bars represent standard deviations. Longitudinal between-group differences in postoperative IOP were evaluated using a linear mixed model, as described in Section 2.4. Abbreviations: IOP, intraocular pressure; OAG, open-angle glaucoma.

Figure 1 illustrates the changes in IOP in both groups over the 12-month postoperative period. At 12 months postoperatively, the mean IOP was 14.02 ± 3.66 mmHg in the two-stent group and 14.03 ± 3.69 mmHg in the three-stent group, showing no significant difference between the two groups (*p* = 0.993).

Table 2 provides a breakdown of the clinical outcomes and changes at 12 months postoperatively. The three-stent group showed a greater unadjusted absolute reduction in IOP from baseline than the two-stent group (−4.00 ± 7.64 mmHg vs. −1.20 ± 4.38 mmHg; unadjusted *p* = 0.047). However, after adjustment for baseline IOP using ANCOVA, the between-group difference was no longer statistically significant (*p* = 0.537).

In the LMM, baseline IOP and concurrent cataract surgery were significant covariates affecting postoperative IOP. The baseline number of topical glaucoma medications was also included as a covariate but was not a significant predictor of postoperative IOP. After adjustment for these factors, there was no significant group effect (F = 0.062, *p* = 0.804) or time-by-group interaction (F = 0.357, *p* = 0.784), indicating that postoperative IOP trajectories did not differ significantly between groups over the 12-month follow-up period. In the exploratory sensitivity analyses, 12-month IOP did not differ significantly between groups in either stand-alone or combined procedure subgroups, whereas the higher baseline medication burden in the three-stent group persisted at 12 months (Supplementary Table S4).

**Figure 2:**
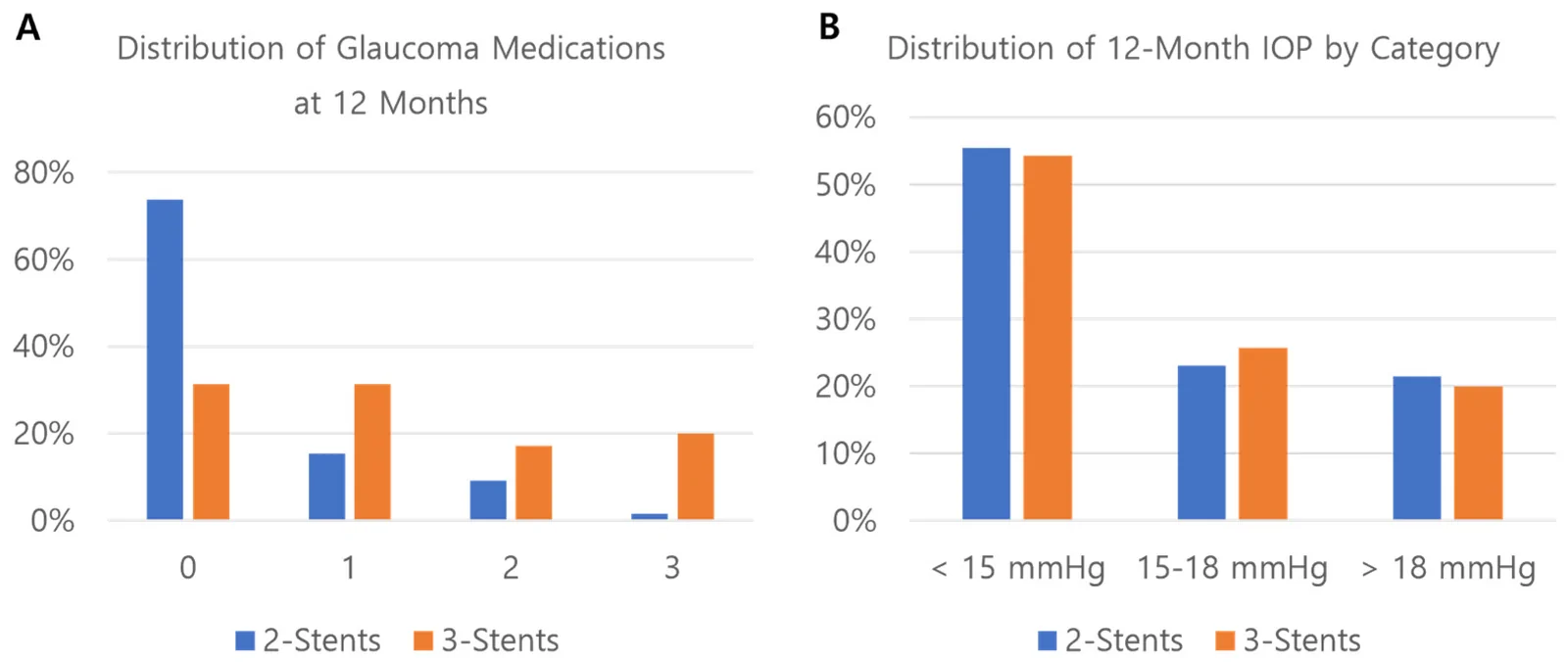
Clinical outcomes regarding medication burden and 12-month IOP distribution. (**A**) Distribution of the number of topical glaucoma medications used. The overall medication count distribution differed significantly between groups (unadjusted *p* < 0.001, chi-squared test), with medication-free eyes comprising 73.8% of the two-stent group and 31.4% of the three-stent group. (**B**) Distribution of 12-month IOP across predefined categories (<15 mmHg, 15–18 mmHg, and >18 mmHg). The distribution did not differ significantly between the groups (unadjusted *p* = 0.953, chi-squared test). Holm-adjusted *p*-values for secondary and exploratory outcomes are provided in Supplementary Table S3.

Table 3 and Figure 2 detail the clinical outcomes regarding medication burden and the distribution of IOP at 12 months. The proportion of eyes with a 12-month IOP < 15 mmHg was similar between the groups (55.4% in the two-stent group vs. 54.3% in the three-stent group; unadjusted *p* = 0.953). Surgical success, as defined in Section 2.3, was achieved in 47 of 65 eyes (72.3%) in the two-stent group and 26 of 35 eyes (74.3%) in the three-stent group, a non-significant difference (*p* = 1.000, Fisher’s exact test; odds ratio 0.90, 95% CI 0.36–2.30). The number of topical glaucoma medications significantly decreased from baseline to 12 months in both the two-stent group (2.02 ± 1.05 to 0.38 ± 0.72, *p* < 0.001) and the three-stent group (2.83 ± 0.87 to 1.26 ± 1.12, *p* < 0.001). The mean reduction was 1.63 ± 1.17 medications in the two-stent group and 1.57 ± 1.52 medications in the three-stent group, with no significant between-group difference (*p* = 0.787). The distribution of the number of topical glaucoma medications at 12 months differed significantly between the groups (unadjusted *p* < 0.001), with medication-free eyes comprising 73.8% of the two-stent group and 31.4% of the three-stent group.

### 3.3. Changes in Visual Field Parameters

Changes in visual field parameters at 12 months are shown in Table 2. The mean change in MD over 12 months was 0.05 ± 3.90 dB in the two-stent group and 0.65 ± 4.33 dB in the three-stent group, with no significant between-group difference (*p* = 0.513). Changes in PSD (0.24 ± 2.82 dB vs. −0.73 ± 2.78 dB, *p* = 0.120) and VFI (−1.84 ± 7.52% vs. 0.52 ± 13.79%, *p* = 0.376) also did not differ significantly between groups.

### 3.4. Safety and Additional Surgeries

Corneal ECD showed a decreasing trend at 12 months postoperatively compared to baseline. However, the reduction did not differ significantly between the groups (214.22 ± 339.68 vs. 249.50 ± 410.10 cells/mm^2^, *p* = 0.659; Table 2). Transient hyphema was observed in 17 eyes (26.2%) in the two-stent group and in 15 (42.9%) in the three-stent group, with no significant between-group difference (*p* = 0.116, Fisher’s exact test); all cases resolved spontaneously without intervention. At the 1-week postoperative visit, early postoperative IOP spikes were observed in 3 eyes (4.6%) in the two-stent group and in 3 (8.6%) in the three-stent group, with no significant between-group difference (*p* = 0.420, Fisher’s exact test). No hemorrhage requiring intervention occurred. At the 3-month postoperative follow-up, gonioscopy confirmed proper stent positioning. Partial occlusion of the stent inlet by the iris was observed in one eye in each group, but no further intervention was necessary. Furthermore, the proportion of eyes requiring additional glaucoma surgery owing to uncontrolled IOP during the follow-up period was 6.2% (4 eyes) in the two-stent group and 14.3% (5 eyes) in the three-stent group, a difference that was not statistically significant (unadjusted *p* = 0.271, Fisher’s exact test; Table 3).

## 4. Discussion

Using a severity-based treatment strategy, this study evaluated 12-month clinical outcomes after the implantation of two stents (iStent inject W) in patients with mild-to-moderate OAG and three stents (iStent infinite) in patients with severe OAG [25]. Both groups showed reductions in IOP and topical medication burden over 12 months, and mean IOP at 12 months was similar between groups despite the higher baseline IOP in the three-stent group [7,9,26]. However, this comparable IOP was achieved with a higher residual medication burden in the three-stent group.

The greater unadjusted IOP reduction observed in the three-stent group (*p* = 0.047) should be interpreted in the context of its higher baseline IOP. After adjustment for baseline IOP using ANCOVA, the between-group difference was no longer significant (*p* = 0.537) [27]. The longitudinal LMM similarly showed no significant group effect or time-by-group interaction, indicating no detectable difference in postoperative IOP trajectories over 12 months after adjustment for measured covariates. These findings should not be interpreted as evidence of equivalence or of an incremental benefit of the third stent.

A possible physiological rationale for the observed IOP control in the severe OAG cohort is that a wider distribution of three stents may increase the likelihood of accessing segments connected to functional distal outflow pathways. Glaucomatous remodeling of the trabecular meshwork extracellular matrix contributes to aqueous outflow resistance, whereas distal aqueous outflow is anatomically variable and segmental [9,28,29,30]. The similar final IOP observed in the three-stent group, despite more advanced glaucoma, supports the clinical feasibility of the three-stent strategy in this cohort [31,32]. This interpretation remains mechanistic and was not directly tested in the present study.

Changes in MD, PSD, and VFI over 12 months did not differ significantly between groups; however, the short follow-up and inter-individual variability, particularly in advanced glaucoma, preclude conclusions regarding functional preservation [33,34,35]. Regarding safety, the numerically higher frequency of transient hyphema in the three-stent group was not statistically significant, and all cases resolved without intervention. Early postoperative IOP spike rates and corneal ECD loss likewise did not differ significantly between groups [36,37,38]. Additional glaucoma surgery was more frequent in the three-stent group, although this difference was not statistically significant. Given the small sample size in severe OAG, the study may have been underpowered to detect differences in failure rates and other secondary outcomes. Overall, these findings suggest an acceptable short-term safety profile for iStent infinite in this severe OAG cohort [9,39].

This study has some limitations. First, its retrospective design introduces potential selection bias and residual confounding. Second, several important assessments were unavailable. In particular, the absence of objective verification with anterior segment optical coherence tomography or ultrasound biomicroscopy means that functionally inadequate patency cannot be ruled out. Third, the 12-month follow-up period is insufficient to assess long-term IOP durability or visual field progression reliably [40,41]. Fourth, concurrent phacoemulsification was performed in a substantial proportion of eyes in both groups (80.0% and 74.3%), and cataract extraction itself may contribute to IOP reduction, particularly in eyes with higher baseline IOP. Although baseline IOP was adjusted for in the ANCOVA, and the LMM also included concurrent cataract surgery and baseline medication burden, the independent effect of stent implantation cannot be fully isolated [42,43]. Fifth, the groups differed substantially in disease severity and treatment allocation, and no severe-OAG cohort receiving two stents was available. Therefore, the present study cannot determine whether three stents are superior to two, or necessary, in severe OAG. Finally, the single-center, single-surgeon design and exclusively Korean population limit generalizability. Prospective, longer-term studies including appropriate comparator groups are warranted.

## 5. Conclusions

In this retrospective severity-stratified cohort, three-stent implantation in severe OAG was associated with 12-month IOP control similar to that observed with two stents in mild-to-moderate OAG, although this was achieved with a greater residual medication burden. These findings support the feasibility of a severity-based management strategy but do not establish that three stents are superior to two, or necessary, in severe OAG. Prospective, long-term, large-scale clinical studies are warranted.

## Figures and Tables

**Table 1:** Baseline clinical characteristics of the study patients.

Characteristics	2-Stent Group (n = 65)	3-Stent Group (n = 35)	*p*-Value
Age (years)	69.96 ± 9.17	69.66 ± 11.26	0.889
Sex (male), n (%)	45 (69.2%)	23 (65.7%)	0.719
Concurrent Cataract Surgery, n (%)			0.511
Stand-alone	13 (20.0%)	9 (25.7%)	
Combined	52 (80.0%)	26 (74.3%)	
BCVA (LogMAR)	0.43 ± 0.33	0.61 ± 0.43	0.023 *
IOP (mmHg)	15.22 ± 3.82	18.03 ± 7.82	0.046 *
Number of Glaucoma Medications	2.02 ± 1.05	2.83 ± 0.87	<0.001 *
Visual Field Parameters			
MD (dB)	−5.83 ± 3.24	−18.97 ± 5.61	<0.001 *
PSD (dB)	4.91 ± 2.86	9.67 ± 2.37	<0.001 *
VFI (%)	89.43 ± 8.83	47.17 ± 20.08	<0.001 *
ECD (cells/mm^2^)	2403.57 ± 448.76	2379.95 ± 412.72	0.800

Data are presented as mean ± standard deviation or number (percentage). BCVA, best-corrected visual acuity; LogMAR, logarithm of the minimum angle of resolution; IOP, intraocular pressure; MD, mean deviation; PSD, pattern standard deviation; VFI, visual field index; ECD, endothelial cell density. * Statistically significant (*p* < 0.05).

**Table 2:** Clinical outcomes and changes at 12 months postoperatively.

Variables	2-Stent Group (n = 65)	3-Stent Group (n = 35)	Unadjusted *p*
IOP (mmHg)			
At 12 months	14.02 ± 3.66	14.03 ± 3.69	0.993
Change from baseline	−1.20 ± 4.38	−4.00 ± 7.64	0.047
Number of Medications			
At 12 months	0.38 ± 0.72	1.26 ± 1.12	<0.001
Reduction from baseline	1.63 ± 1.17	1.57 ± 1.52	0.787
Visual Field Parameters			
MD at 12 months (dB)	−5.78 ± 4.74	−18.33 ± 8.18	<0.001
Change in MD (dB)	0.05 ± 3.90	0.65 ± 4.33	0.513
PSD at 12 months (dB)	5.15 ± 3.38	8.94 ± 3.11	<0.001
Change in PSD (dB)	0.24 ± 2.82	−0.73 ± 2.78	0.120
VFI at 12 months (%)	87.59 ± 11.18	47.69 ± 25.93	<0.001
Change in VFI (%)	−1.84 ± 7.52	0.52 ± 13.79	0.376
ECD (cells/mm^2^)			
At 12 months	2189.35 ± 489.23	2130.45 ± 333.15	0.524
Reduction from baseline	214.22 ± 339.68	249.50 ± 410.10	0.659

Data are presented as mean ± standard deviation. IOP, intraocular pressure; MD, mean deviation; PSD, pattern standard deviation; VFI, visual field index; ECD = endothelial cell density. Unadjusted *p*-values are shown for unadjusted between-group comparisons. For the primary efficacy analysis of the 12-month change in IOP, the baseline-adjusted *p*-value was obtained using ANCOVA and is reported in the text and Supplementary Table S1. Holm-adjusted *p*-values for secondary and exploratory outcome comparisons are provided in Supplementary Table S3.

**Table 3:** 12-month IOP distribution, medication burden, surgical success, and additional glaucoma surgery at 12 months postoperatively.

Outcomes	2-Stent Group (n = 65)	3-Stent Group (n = 35)	Unadjusted *p*
12-Month IOP Distribution, n (%)			0.953
<15 mmHg	36 (55.4%)	19 (54.3%)	
15–18 mmHg	15 (23.1%)	9 (25.7%)	
>18 mmHg	14 (21.5%)	7 (20.0%)	
Success rate at 12 months, n (%)	47 (72.3%)	26 (74.3%)	1.000
Number of Medications Used, n (%)			<0.001
0	48 (73.8%)	11 (31.4%)	
1	10 (15.4%)	11 (31.4%)	
2	6 (9.2%)	6 (17.1%)	
3	1 (1.5%)	7 (20.0%)	
Additional Glaucoma Surgery, n (%)			0.271
No	61 (93.8%)	30 (85.7%)	
Yes	4 (6.2%)	5 (14.3%)	

Data are presented as number (percentage). IOP = intraocular pressure. Unadjusted *p*-values are shown. The chi-squared test was used for the 12-month IOP category distribution and medication count distribution; Fisher’s exact test was used for the success rate (defined in Section 2.3) and additional glaucoma surgery. Holm-adjusted *p*-values for the secondary and exploratory outcome family are provided in Supplementary Table S3.

## Data Availability

The data presented in this study are available on request from the corresponding author. The data are not publicly available due to privacy and ethical restrictions regarding patient medical records.

## Supplementary Materials

The following supporting information can be downloaded at: https://www.mdpi.com/article/10.3390/jcm15166396/s1, Table S1: Parameter estimates from the analysis of covariance (ANCOVA) model for the 12-month change in intraocular pressure (IOP), adjusted for baseline IOP; Table S2: Fixed-effects tests and parameter estimates from the linear mixed model for longitudinal intraocular pressure during the 12-month postoperative follow-up; Table S3: Holm-adjusted *p*-values for secondary/exploratory outcome comparisons; Table S4: Exploratory stratified analysis of intraocular pressure and medication outcomes according to concurrent cataract surgery status.

## Abbreviations

The following abbreviations are used in this manuscript:

AS-OCT	Anterior Segment Optical Coherence Tomography
BCVA	Best-Corrected Visual Acuity
ECD	Endothelial Cell Density
IOP	Intraocular Pressure
LogMAR	Logarithm of the Minimum Angle of Resolution
MD	Mean Deviation
MIGS	Minimally Invasive Glaucoma Surgery
OAG	Open-Angle Glaucoma
OVD	Ophthalmic Viscoelastic Device
PSD	Pattern Standard Deviation
VFI	Visual Field Index
