# Supplementary material for: Twelve-Month Outcomes of iStent Infinite in Severe Open-Angle Glaucoma Compared with iStent Inject W in Mild-to-Moderate Disease
Source: J Clin Med. 2026 Aug 19;15(16):6396. doi: 10.3390/jcm15166396 (PMC13513176; doi:10.3390/jcm15166396)
Supplement: Supplementary file 1 [file jcm-15-06396-s001.zip › jcm-4447372-supplementary.pdf]

Supplementary Tables

Table S1. Parameter estimates from the analysis of covariance (ANCOVA) model for the 12-month change in intraocular pressure (IOP), adjusted for baseline IOP.

Parameter	B	SE	t	p	95% CI Lower	95% CI Upper
Intercept	10.874	1.304	8.342	<0.001	8.283	13.466
Baseline IOP	-0.825	0.065	-12.716	<0.001	-0.954	-0.696
Group (three-stent vs. two-stent)	0.485	0.782	0.620	0.537	-1.070	2.039

N = 100 eyes. The dependent variable was the 12-month change in IOP (mmHg) from baseline. The two-stent group was used as the reference category for Group. B, unstandardized regression coefficient; SE, standard error; CI, confidence interval.

Table S2. Fixed-effects tests and parameter estimates from the linear mixed model for longitudinal intraocular pressure during the 12-month postoperative follow-up.

Panel A. Type III tests of fixed effects

Fixed effect	Numerator df	Denominator df	F	p-value
Group	1	338.241	0.062	0.804
Time	3	166.116	0.419	0.739
Group × Time	3	166.116	0.357	0.784
Concurrent cataract surgery	1	332.173	18.862	<0.001
Baseline IOP	1	332.068	20.743	<0.001
Baseline number of glaucoma medications	1	330.989	0.860	0.354

Panel B. Fixed-effects parameter estimates

Parameter	B	SE	df	t	p	95% CI Lower	95% CI Upper
Intercept	13.282	1.084	272.810	12.256	<0.001	11.149	15.416
Group (three-stent vs. two-stent)	0.418	0.781	91.824	0.535	0.594	-1.133	1.968
Time (1 month vs. 12 months)	0.974	0.884	169.676	1.102	0.272	-0.771	2.718
Time (3 months vs. 12 months)	0.553	0.818	173.023	0.675	0.500	-1.063	2.168
Time (6 months vs. 12 months)	-0.079	0.774	166.453	-0.102	0.919	-1.606	1.448
Time (1 month) × Group	-0.895	1.168	169.676	-0.767	0.444	-3.200	1.410
Time (3 months) × Group	-0.494	1.081	173.023	-0.457	0.648	-2.627	1.640
Time (6 months) × Group	0.138	1.019	166.242	0.135	0.893	-1.875	2.150
Concurrent cataract surgery	-2.073	0.477	332.173	-4.343	<0.001	-3.012	-1.134
Baseline IOP	0.156	0.034	332.068	4.554	<0.001	0.089	0.224
Baseline number of glaucoma medications	-0.193	0.208	330.989	-0.927	0.354	-0.602	0.216

N = 100 eyes (400 observations across four postoperative visits: 1, 3, 6, and 12 months). The dependent variable was postoperative IOP (mmHg). Fixed effects included group, time, group-by-time interaction, concurrent cataract surgery, baseline IOP, and baseline number of glaucoma medications. Reference categories were the two-stent group for Group and 12 months for Time. Panel A presents Type III omnibus tests of fixed effects. Panel B presents individual fixed-effect parameter estimates. B, unstandardized regression coefficient; SE, standard error; df, denominator degrees of freedom; CI, confidence interval.

Table S3. Holm-adjusted p-values for secondary/exploratory outcome comparisons.

Outcome	Unadjusted p-value	Holm-adjusted p-value
Distribution of number of glaucoma medications at 12 months	<0.001	0.001
Change in PSD	0.120	0.960
Additional glaucoma surgery	0.271	1.000
Change in VFI	0.376	1.000
Change in MD	0.513	1.000
ECD reduction	0.659	1.000
Reduction in number of glaucoma medications	0.787	1.000
12-month IOP distribution	0.953	1.000
Success rate at 12 months, n (%)	1.000	1.000

Unadjusted and Holm-adjusted p-values are presented for nine secondary/exploratory between-group comparisons. P-values were adjusted using the Holm–Bonferroni procedure. The chi-squared test was used for the medication-count distribution and the 12-month IOP distribution; Fisher's exact test was used for surgical success and additional glaucoma surgery. Between-group comparisons of changes in the number of glaucoma medications, MD, PSD, VFI, and ECD correspond to those reported in the main manuscript. The primary IOP analysis was evaluated separately and was not included in this multiplicity-adjustment family. IOP, intraocular pressure; MD, mean deviation; PSD, pattern standard deviation; VFI, visual field index; ECD, endothelial cell density.

Table S4. Exploratory stratified analysis of intraocular pressure and medication outcomes according to concurrent cataract surgery status.

**Stand-alone procedures**			
**Outcome**	**2-Stent Group (n = 13)**	**3-Stent Group (n = 9)**	**p-value**
Baseline IOP (mmHg)	17.62 ± 3.98	20.67 ± 4.72	0.119
IOP at 12 months (mmHg)	15.00 ± 3.16	15.11 ± 4.79	0.871
12-month IOP change (mmHg)	-2.62 ± 4.25	-5.56 ± 5.50	0.224
Baseline number of medications	2.62 ± 0.85	3.22 ± 0.67	0.040
Number of medications at 12 months	0.31 ± 0.85	1.56 ± 1.32	0.007
12-month medication reduction	2.31 ± 0.94	1.67 ± 1.70	0.080
**Combined phacoemulsification**			
**Outcome**	**2-Stent Group (n = 52)**	**3-Stent Group (n = 26)**	**p-value**
Baseline IOP (mmHg)	14.62 ± 3.63	17.12 ± 8.54	0.435
IOP at 12 months (mmHg)	13.77 ± 3.78	13.65 ± 3.23	0.835
12-month IOP change (mmHg)	-0.85 ± 4.39	-3.46 ± 8.29	0.257
Baseline number of medications	1.87 ± 1.05	2.69 ± 0.90	0.004
Number of medications at 12 months	0.40 ± 0.69	1.15 ± 1.05	0.004
12-month medication reduction	1.46 ± 1.17	1.54 ± 1.49	0.655

Data are presented as mean ± standard deviation. Between-group comparisons were performed using an independent t-test or Mann–Whitney U test, as appropriate. These subgroup analyses were exploratory and were not adjusted for multiple comparisons. IOP, intraocular pressure.
